# Graphene-based flexible antenna for wearable and biomedical devices

**DOI:** 10.1038/s41598-026-58473-3

**Published:** 2026-07-10

**Authors:** Nada M. Sayed, Ayad Shohdy, Ahmed M. Montaser

**Affiliations:** 1 Faculty of Technology and Education, Sohag University, Sohag, Egypt; 2 Faculty of Engineering, Sohag University, Sohag, Egypt

**Keywords:** Wearable antenna, Flexible materials, Graphene, ISM band, Biomedical applications, Wireless communication, Tumor detection, Engineering, Physics

## Abstract

Flexible antenna design is described in this study, prototyping of a flexible graphene-based antenna for data transmission from wearable arm and abdomen imaging equipment over a 5G network. The antenna uses an 18-μm-thick graphene thin sheet for the conductive and ground radiating patch. The proposed design operates in the from 25.2–40.2 GHz frequency band and it based on the radiation patch fractal structure which allowed for adequate antenna flexibility. The invention is appropriate for wearable applications because the patch was constructed on a flexible polyamide substrate that was 1.575 mm thick. In terms of the radiation pattern, gain, and antenna reflection coefficient, the proposed design is studied and analyzed. Additionally, a time-domain signal analysis between two antennas as transmitter and receiver was carried out to mimic wearable device real-time communication. A 3D modeling and analysis of a flexible 5G antenna for communication in the arm to assess its basic electromagnetic properties is introduced. Also the design was used with abdomen in order to replicate actual biological settings, detect and track tumor growth and dissemination throughout the body. The simulation results showed that the antenna shows simulated sensitivity to tumor-induced dielectric changes, particularly in its advanced stages.

## Introduction

In the contemporary era, communication systems have undergone remarkable progress, achieving unprecedented developments in data transmission technologies. The attainment of extremely high data rates, reduced latency, superior network performance, reliable communication links, data consistency, extensive network capacity, greater user adaptability, and uniform service availability has revolutionized wireless communication technologies^1,2^.

. As the prevalence of both communicable and non-communicable diseases (NCDs) rises, maintaining patient data and healthcare systems as a whole becomes increasingly important. A round all the worldwide a 74%^3^ of deaths are caused by NCDs, which include long-term respiratory disorders, diabetes, cancer, and heart disease. Of these fatalities 85% take place in nations with low and moderate incomes^4^.

Wearable technology will enable sophisticated communication in the biomedical industry in the next years, offering cost-effective and efficient solutions. The key applications are in telemedicine and smart health care systems for health monitoring. For wearable antennas operating in the ISM band, rectangular and circular patches with various slot types are ideal antenna designs because they improve impedance matching and manage surface waves^5–8^. Data collection and monitoring for multiple patients simultaneously was a critical task. Consequently, efficient tracking of health metrics such as blood pressure, heart rate, stress, oxygen, and degree of fitness, is made possible by wearable technology^9^. Rarely does the body’s tissue burden affect the antenna’s effectiveness, especially wearable or implantable ones, by altering their performance through absorption and reflection, causing frequency shifts, reduced efficiency, and impedance changes, as tissues act as a lossy , variable medium with different dielectric properties depending on fat, muscle and skin composition^10^.

Rigid substance attached to a body causes microscopic fissures when bent, which reduces the antenna’s bandwidth and gain. In order to get over this problem, the body-worn antenna ought to be composed of flexible, not stiff, material^11^. Conventional conductive materials like copper (Cu) and aluminum (Al) are commonly employed owing to their excellent electrical conductivity and widespread availability. Nevertheless, issues such as susceptibility to corrosion, relatively high fabrication costs, and limited mechanical resilience have encouraged the investigation of alternative conductive materials^12^ The electrical conductivity of the radiating element directly affects antenna characteristics, as higher conductivity reduces ohmic losses and enhances radiation efficiency. Although metallic conductors continue to dominate antenna fabrication, their practical limitations have motivated research into novel materials that provide a suitable compromise between conductivity, flexibility, durability, and economic feasibility.

To address the aforementioned challenges, recent studies have investigated advanced material platforms including polymers, metamaterials, and nanocomposite structures, which provide enhanced flexibility, reduced weight, and improved compatibility with wearable technologies^13^. Nevertheless, determining the most effective combination of substrate and conductive materials for achieving optimal antenna performance remains an open research issue^14^. In parallel, Kapton, a flexible polyimide-based film, demonstrates remarkable thermal endurance and mechanical reliability, which makes it highly appropriate for conformal and wearable implementations^15^. Furthermore, Kapton has shown promising sensing capabilities in flexible detection systems, particularly when integrated with graphene-based materials systems for health monitoring^16^. Compared with traditional metallic conductors such as copper, silver, and gold, carbon-based nanomaterials offer competitive electrical performance along with improved adaptability for next-generation antenna structures^17^. In addition, carbon- and graphene-derived materials exhibit high sensitivity to electromagnetic absorption, which further enhances their suitability for sensing applications, telemedicine and smart health care systems for health monitoring^18^.

More designs in literature are provided which related to the present study. A. Riaz et al. presented a flexible graphene patch antenna designed for wearable wireless communication systems^3^ .The antenna was fabricated on a flexible polymer substrate and analyzed using full-wave electromagnetic simulation tools. The results demonstrated stable impedance matching and radiation characteristics under various bending conditions, confirmed the suitability of graphene as a reliable radiating material for flexible and wearable antenna designs. Al-Gburi et al. designed a flexible graphene-based antenna for wearable biomedical monitoring applications^19^ .The antenna performance was evaluated in proximity to a multilayer human tissue model consisting of skin, fat, and muscle layers. Specific absorption rate (SAR) analysis was conducted, and the results confirmed compliance with IEEE safety standards while maintaining stable radiation characteristics. Similarly Babu, K. Vasu, et al. introduce a design antenna for high-performance terahertz 6G communication^20^ . The antenna was simulated using CST Microwave Studio and analyzed under different bending scenarios. The results indicated that graphene-based antennas can achieve lower SAR values compared to traditional metallic antennas due to their thin structure and distributed surface currents. However, the antenna operated only in lower microwave frequency bands. Abohmra, et al. propose a flexible and wearable graphene-based terahertz antenna for body-centric applications^21^. The antenna exhibited compact dimensions and acceptable gain performance when placed close to the human body. The authors emphasized that graphene-based antennas experience reduced performance degradation under mechanical deformation compared to conventional metallic antennas. John, D. M., et al. present a flexible antennas for a Sub-6 GHz 5G band^22^ .The antenna achieved compact size, acceptable gain, and stable performance under bending conditions. Although the design targeted wearable communication systems, limited emphasis was placed on detailed biomedical safety and SAR analysis. In another study, Ibanez-Labiano, Isidoro, et al. present a graphene-based soft wearable antennas^23^.The antenna was designed on a flexible substrate and evaluated using a simplified human body phantom. The results showed satisfactory impedance matching and radiation efficiency; however, the use of a single-layer tissue model may not accurately represent realistic biomedical environments.

The aim of this work is to present and design a novel graphene-based flexible antenna for Ka-band 5G connection for wearable and biomedical devices. The designed antenna will enable the wearable, portable device to monitor the patient in real time and transmit the data which it gathers via the 5G frequency band to the desired destination for further analysis. The antenna has a graphene radiating patch on a Kapton polyimide substrate. The design was used with arm and abdomen in order to replicate actual biological settings, detect and track tumor growth and dissemination throughout the body. The simulation results showed that the antenna could identify changes brought on by the tumor, particularly in its advanced stages. The proposed design and its analysis are presented in details in the next sections.

## Antenna design

**Fig. 1:**
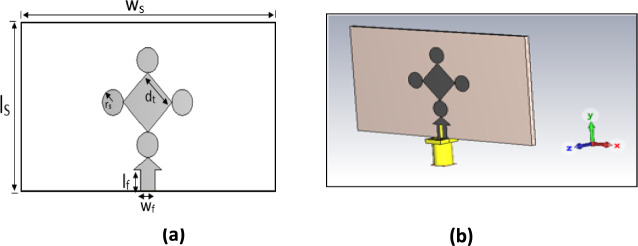
The proposed flexible antenna, (**a**) front view, (**b**) 3D simulated view.

The antenna was designed using the CST microwave studio software. The construction was made flexible and robust using Kepton HN polyamide^3^, a flexible substrate that is 1.575 mm thick and has a relative dielectric of 3.5 F/m. Micro strip feeding was used to feed the antenna; the feed line’s impedance is matched to the antenna’s input impedance. This method required little spurious radiation and was simple to construct. Improving antenna gain, and impedance matching were its main goals^24^ . The radioactive patch of the extremely basic suggested antenna is made up of four equal circular patches positioned at a square-shaped antenna’s corners. Figure 1a displays the front view of the antenna. An 18 µm thin graphene film with a relative permittivity of 100.92 is used to build the antenna for the conductive patch and ground. Figure 1b shows the simulated suggested design.

The suggested design achieves over a 15 GHz wideband. Instantaneous spectrum access was made possible by the architecture, allowing for the use of a single antenna rather than several. Frequently used flexible wearable portable devices benefited greatly from the structure’s ability to withstand the most severe situations. A thorough overview of the design dimensions is given in Table 1.

**Table 1:** The proposed flexible antenna design parameters.

Description	Parameter	Value (mm)
Radius of the circle	r_s_	3
Graphene patch thickness	h_g_	0.018
Substrate thickness	h_s_	1.575
Square side length	d_t_	7
Patch length	L_s_	40
Patch width	W_s_	75
Feed line length	l_f_	5
Feed line width	w_f_	2

## Simulation results

The proposed antenna’s simulation is predicated on the time-domain finite integration technique (FIT), which is performed using CST Microwave Studio. The proposed patch is based on graphene and to preserve the best possible antenna performance, it is incorporated into the fractal geometry. In order to analyze the antenna’s gain and return loss, the antenna shape was optimized. The following is a description of the design’s important performance parameters.

### Return loss (S11)

**Fig. 2:**
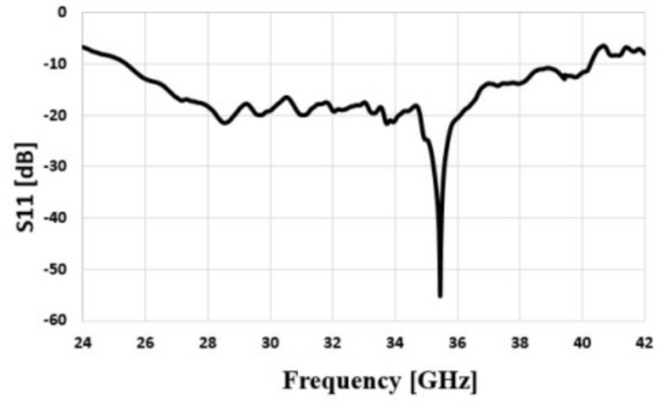
S11 parameter of the proposed flexible antenna.

The return loss of the antenna is shown by the scattering parameters (S-parameters) graph in Fig. 2. When the antenna is energized, some of the input power was lost or reflected in the surroundings, and the rest was sent. The quantity of reflected power in the antenna construction is indicated by the return loss value. Numerous factors, both internal to the antenna structure and external to it, contributed in this reflection. Impedance mismatches and discontinuities at connections are the two primary causes which increase the return loss. The return loss of the antenna should be less than -10 dB for best results. The simulated return loss of the recommended antenna had the lowest value of -43.2 dB at the resonance frequency of 35.4 GHz, as shown in Fig. 2. The wide operating bandwidth of the antenna was roughly 15 GHz, ranging from 25.2 GHz to 40.2 GHz. Return loss has a practical effect on telemedicine since a lower return loss value will result in a higher power transmission to the telemedicine system’s receiving end.

### Radiation pattern and gain

**Fig. 3:**
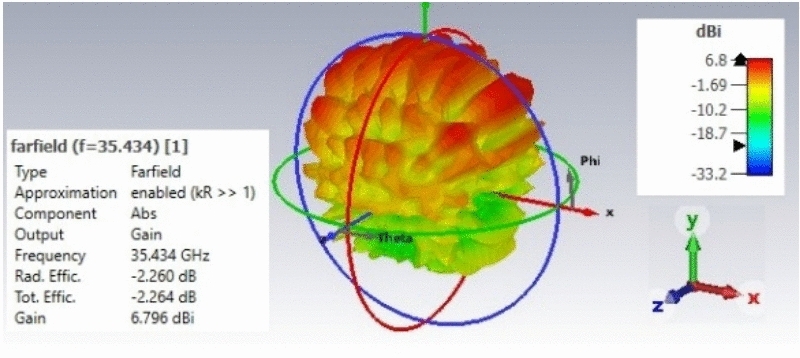
3D radiation pattern of the proposed flexible antenna.

When compared to a traditional antenna, an antenna’s gain indicates how much it can radiate in any direction. For instance, an antenna would radiate uniformly in all directions if it could be constructed exactly spherical. The antenna’s transmitting power in a particular direction increases with its gain. In a similar vein, antenna gain compares the average antenna power density across all directions to the antenna’s power density in the direction of highest radiation. When the antenna’s gain is increased, the radiation beam travels farther and the concentration of radiation in a certain direction is improved. Figure 3 shows the three-dimensional radiation pattern that illustrates the gain of the proposed flexible antenna at frequency 35.434 GHz. As seen in the figure, the antenna’s gain is 6.796 dBi at this frequency.

### Gain characteristics of the proposed antenna

**Fig. 4:**
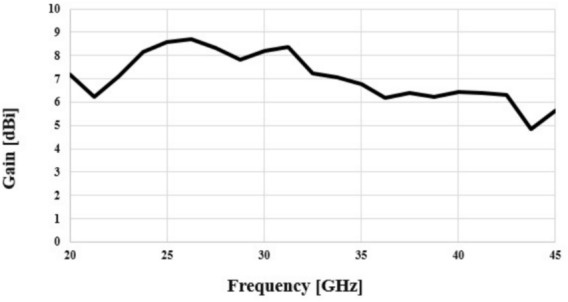
Gain versus frequency of the proposed antenna**.**

The gain performance of the proposed antenna was evaluated over the frequency range from 20 to 45 GHz, as illustrated in Fig. 4. The results demonstrate that the antenna maintains a relatively stable gain response across the investigated millimeter-wave band, with gain values ranging approximately from 5 dBi to 8.7 dBi. The maximum gain is achieved around 25–26 GHz, while the antenna still preserves acceptable radiation characteristics at higher frequencies. At frequency of 35.4 GHz, the antenna exhibits a gain of approximately 6.7 dBi, indicating efficient radiation capability and stable performance suitable for high-frequency biomedical and wireless communication applications. The obtained gain behavior confirms the effectiveness of the proposed antenna design in providing reliable performance within the desired operating band.

### Radiation efficiency performance of the proposed antenna

**Fig. 5:**
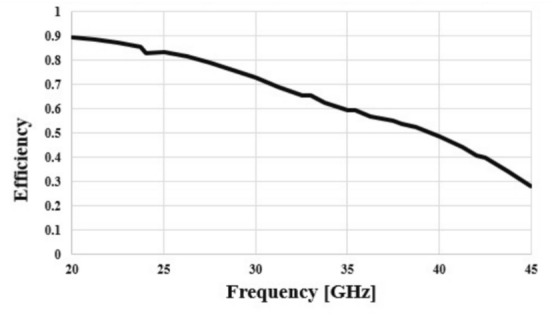
Radiation efficiency versus frequency of the proposed antenna.

The radiation efficiency of the proposed antenna was analyzed over the frequency range from 20 to 45 GHz, as presented in Fig. 5. The obtained results indicate that the antenna achieves high efficiency performance across the investigated millimeter-wave band, demonstrating its capability to effectively convert the accepted input power into radiated electromagnetic energy with minimal losses. At lower frequencies, the antenna exhibits good efficiency values exceeding 90%, which confirms the low conductive and dielectric losses of the proposed structure. As the operating frequency increases, a gradual reduction in efficiency is observed due to the higher propagation and material losses typically associated with millimeter-wave frequencies. Nevertheless, the antenna maintains acceptable radiation efficiency throughout the operating band. The antenna achieves an average efficiency of approximately 70% over the operating band, which reflects stable radiation behavior and good impedance performance at the desired frequency band. This efficiency level confirms that the proposed antenna is capable of maintaining reliable electromagnetic radiation characteristics suitable for high-frequency biomedical sensing and wireless communication applications. The obtained results demonstrate that the proposed design successfully balances compactness, gain performance, and radiation efficiency within the targeted millimeter-wave spectrum.

### Surface current distribution

**Fig. 6:**
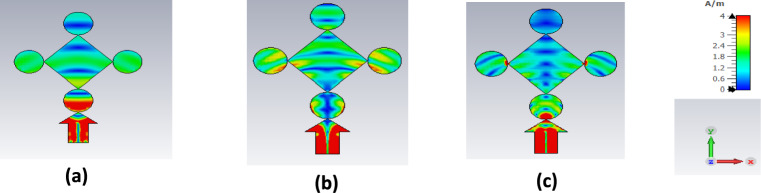
Surface current distribution of the proposed antenna at (**a**) 25.2 GHz, (**b**) 35.4 GHz, and (**c**) 40.2 GHz**.**

The surface current distribution of the proposed antenna was analyzed at 25.2 GHz Fig. 6a, 35.4 GHz Fig. 6b, and 40.2 GHz Fig. 6c, to investigate its radiation characteristics. At 25.2 GHz, the current is mainly concentrated at the feeding line with a moderate spread across the radiating patch, indicating initial excitation of the antenna. At 35.4 GHz, a significantly stronger and more uniform current distribution is observed along the patch and its edges, confirming optimal resonance, efficient radiation, and proper impedance matching at the desired operating frequency. At 40.2 GHz, the current remains concentrated near the feed region with a noticeable distribution over the structure, although it appears less uniform compared to 35.4 GHz. Overall, the surface current behavior across these frequencies demonstrates effective excitation of the antenna, with the best performance achieved at 35.4 GHz due to the enhanced current intensity and symmetric distribution.

## Time Domain communication analysis

**Fig. 7:**
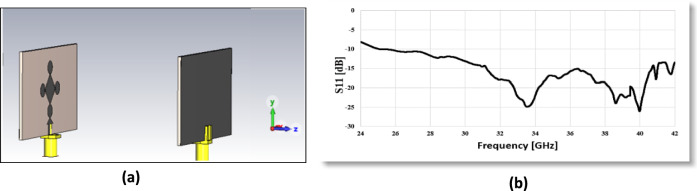
Time domain analysis, (**a**) front-to-front antennas, (**b**) S11 parameter.

A front-to-front antenna model that replicated the two flexible 5G antenna prototypes and looked at time domain communications is shown in Fig. 7a. The return loss value of -24.9 dB at frequency of 33.6 GHz indicates that there was enough power to transfer over the antennas, as shown in Fig. 7b, with wider bandwidth. As a result, our architecture is well suited for telemedicine.

## Interaction between human body and the proposed antenna

Electronic wireless communication devices are used in the current medical industry to transmit patient vital signs to health experts in a timely and dependable manner. Wireless gadgets are also used by health trainers to monitor individuals’ fitness levels. These devices operate inside a wireless body area network (WBAN), a human-centered communication network that serves as a sensor network for the transmission and collecting of critical data^25^. Multi-band antennas are required for ON/OFF communications in WBAN systems. The performance of WBAN antennas can be altered by a variety of conditions, including human proximity. The primary factors to consider while selecting the antenna form and design technique are impedance matching, radiation characteristics, SAR value, and polarization^26^. Compared to standard antennas, wearable antenna design is more challenging^27–33^.

A multilayer tissue model of the human hand is utilized for implanted device modeling, wearable antennas, SAR testing, and arm imaging. Skin, fat, muscle, and bone make up the human arm model. Depending on the frequency employed, each tissue model has a distinct layer as well as varying relative permeability and conductivity. The layer’s electrical characteristics are examined in relation to its length. In 3D electromagnetic simulations, the cognitive characteristics of this layer are frequently employed to generate basic layered phantoms^34,35^. Analysis of antenna performance on the human arm: The antenna’s radiation properties, gain, and frequency detuning all alter when it is put on the human arm. Human tissues are taken into account as multilayer, lossy dielectric medium in electromagnetic study of wave propagation inside the human body. Because of the distance between the device and the skin, fat, muscle, and bone in wearable applications, electromagnetic waves are transmitted via the air. Wave attenuation, transmission, and reflection are all impacted by the distinct dielectric characteristics of each layer. As a result, the properties of the surrounding body tissues may have a major impact on antenna performance^11^.

Because of the surface’s natural curvature, flexible micro strip antennas can be installed or worn on a range of human body parts, including arms, wrists, legs, necks, shoulders, and more. Antennas may adapt to various body shapes thanks to their flexibility. Antennas may be shaped to fit various body parts with varying bending angles thanks to their flexibility. Textile-based micro strip antennas may be suitable options for this use. In these antennas, flexible textile material is used in place of the hard dielectric substrate. Researchers studying wireless communication around the world are very interested in these kinds of textile-based flexible antennas, which has led to a large number of research publications on the subject.

### Interaction with human hand

**Fig. 8:**
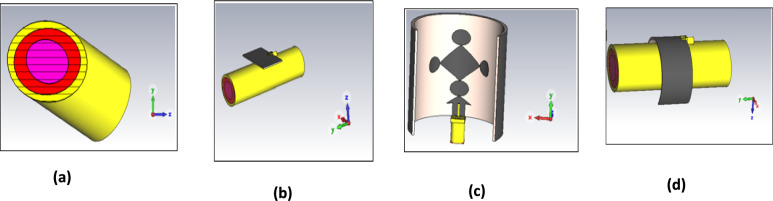
Interaction with human hand, (**a**) human hand, (**b**) the antenna was placed flat, (**c**) curved without hand, (**d**) curved with hand.

A human arm model with three biological layers—skin, fat, and muscle—with corresponding thicknesses of 2 mm, 11 mm, and 14 mm^36^ was covered with a composite layer made of graphene and polyimide, as illustrated in Fig. 8a. Based on direct measurements of a human subject, the arm was modeled as an oval cross-section with an average major and minor radius of 27 mm and 20 mm, respectively. Table 2 lists the electrical and thermal characteristics at the operated frequency as well as the thickness of each hand layer.

**Table 2:** Thickness of each layer in the hand and abdomen, as well as electrical and thermal characteristics^37–39^.

Tissue	Thickness (mm)	${\boldsymbol{\varepsilon}}\mathrm{r}$	${\boldsymbol{\sigma}}$ (S/m)	${\boldsymbol{\rho}}$ (Kg/m^3^)	K (W/m/k)	C (J/Kg/k)
Muscle	14	21.442	38.07	1090	0.49	3421
Fat	11	3.558	1.9303	911	0.21	2348
Skin	2	16.458	29.342	1109	0.37	3391
Tumor	-	50	2.39	997	0.64	3978

**Fig. 9:**
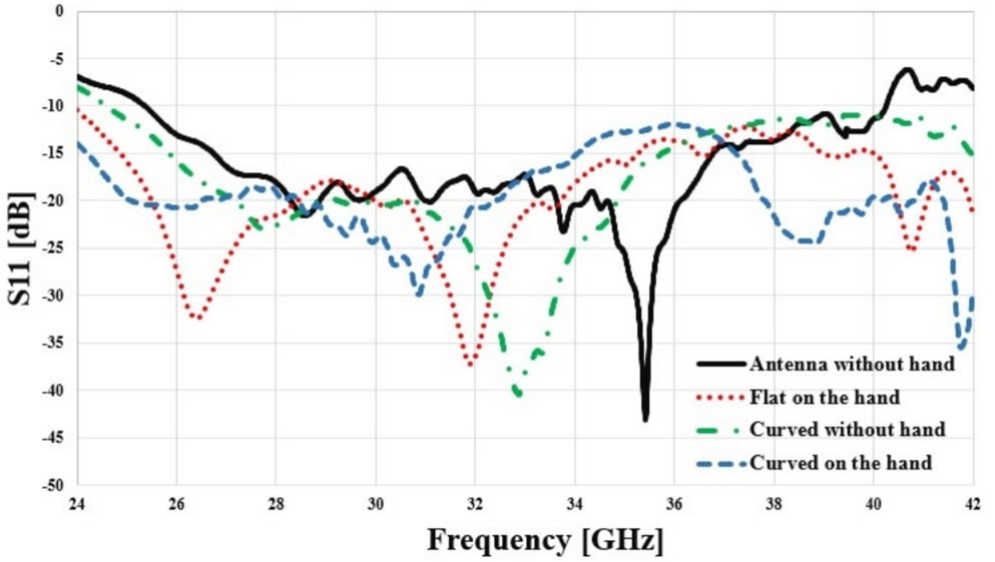
S11 parameter of the interaction with human hand.

The antenna performance was tested through three main stages as presented in Fig. 8, in order to evaluate its performance under different conditions that simulate realistic usage scenarios. The simulated results are provided in Fig. 9. The antenna design result in free space without the presence of the hand in is also included in the figure for comparison. By comparing the obtained results from the three testing stages, it can be observed that both the resonant frequency and the reflection coefficient S11 were significantly affected by the testing environment and the surface on which the antenna was placed**.**

In the first stage, when the antenna was placed flat on the human arm as shown in Fig. 8b. The results indicated the reflection coefficient decreased to -37.115 dB at a resonant frequency of 31.902 GHz, as a result of the biological tissues (skin, fat, and muscle), which absorb part of the electromagnetic energy and alter the effective dielectric constant surrounding the antenna.

In the second stage, applying curvature without the arm as shown in Fig. 8c, led to a shift in the resonant frequency to 32.862 GHz with an improved reflection coefficient -40.616 dB. This improvement is mainly attributed to the effect of the bending shape on the surface current distribution within the antenna.

Finally, in the third stage, when the antenna was tested on the actual arm under bending conditions as shown in Fig. 8d, the reflection coefficient slightly decreased to -30.902 dB with a minor frequency shift to 30 GHz, due to full electromagnetic interaction between the antenna and the biological medium. These results clearly demonstrate that the antenna’s performance is strongly influenced by both the biological environment and the geometrical bending. Therefore, studying the antenna behavior under realistic body-related conditions is crucial for its use in biomedical applications. This pronounced interaction suggests that graphene can effectively couple with biological tissues, enhancing signal detection and transmission at millimeter-wave frequencies.

Furthermore, the results confirm that the multilayer structure of skin, fat, and muscle plays a crucial role in determining the overall field distribution and energy penetration depth. The presence of the graphene–polyimide layer enhances the electromagnetic field concentration near the tissue interface, which could be advantageous for applications such as non-invasive bio sensing, localized hyperthermia treatment, and real-time physiological monitoring. Overall, these results show that graphene-based materials have the potential to enhance the functionality of biomedical devices that use electromagnetic interaction, providing a possible route toward more sophisticated wearable and implantable health systems.

### Interaction with human abdomen

The human abdomen, which has three primary layers skin, fat, and muscle with thicknesses of 2 mm for the skin, 11 mm for the fat, and 27 mm for the muscle, was also covered with a layer made of graphene and polyimide.

**Fig. 10:**
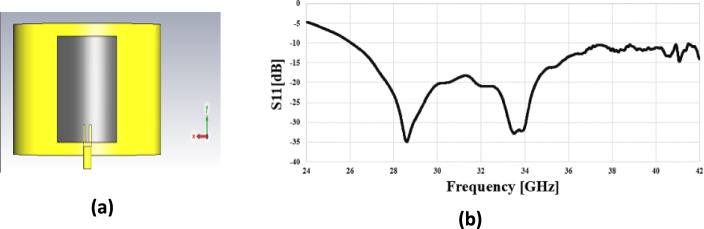
(**a**) The proposed flexible antenna integrated onto a human abdomen, (**b**) S11 parameter.

The abdomen was modeled as an elliptical cross-section with an average major and minor radius of 60 mm and 40 mm respectively, determined from direct measurements of a human subject. The abdominal region was modeled and represented as an elliptical shape to simulate the realistic geometry of the human abdomen. The composite layer composed of graphene and polyimide was integrated onto a human abdomen as shown in Fig. 10a.

Figure 10b illustrate the impact of the human abdomen on the graphene–polyimide film and proving its electromagnetic performance vs. frequency. A reflection coefficient (S11) of roughly –35 dB and –32.964 dB was found in the collected results at a frequency of 28.6 GHz and 33.57 GHz respectively, these findings confirm the proposed design’s potential for wearable and biomedical sensing applications, particularly for non-invasive physiological indicator skin monitoring.

Cancer is regarded as one of the deadly illnesses. The basic building blocks of the body are cells, which multiply and split to create new cells when the body needs them. When cells get too old or damaged, they typically die and are replaced by new ones. When genetic alterations disrupt this regular mechanism, cancer develops. Uncontrollably growing cells develop into a mass known as a tumor. A tumor Assistant editor Gustavo Calico was in charge of reviewing the manuscript and approving its publication. is either benign or malignant; a malignant tumor may develop and spread to other parts of the body. A benign tumor can grow but won’t spread^40,41^.

One of the hardest malignancies to treat is abdominal cancer, which includes tumors of the stomach, colon, liver, and surrounding soft tissues. Because of their special physical and chemical characteristics—high surface area, photo thermal conversion efficiency, catalytic activity, and biocompatibility—nanomaterial’s like graphene and polyimide-based composites have drawn a lot of attention in recent years for cancer diagnosis and treatment. When polyimide particles and graphene sheets are combined, a hybrid nanostructure is created that can function as a photo thermal agent and a drug carrier at the same time, improving the local destruction of tumor tissue in the abdominal area. A polyimide-graphene composite may interact with surrounding healthy tissue and malignant tissue in an abdominal region containing tumor tissue through a number of mechanisms:

#### Tumor targeting and penetration

**Fig. 11:**
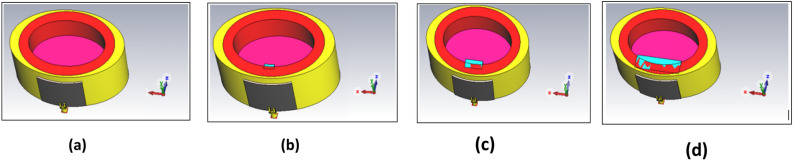
Tumor growing and penetration, (**a**) No tumor, (**b**) Stage I, (**c**) Stage II, (**d**) Stage III.

**Fig. 12:**
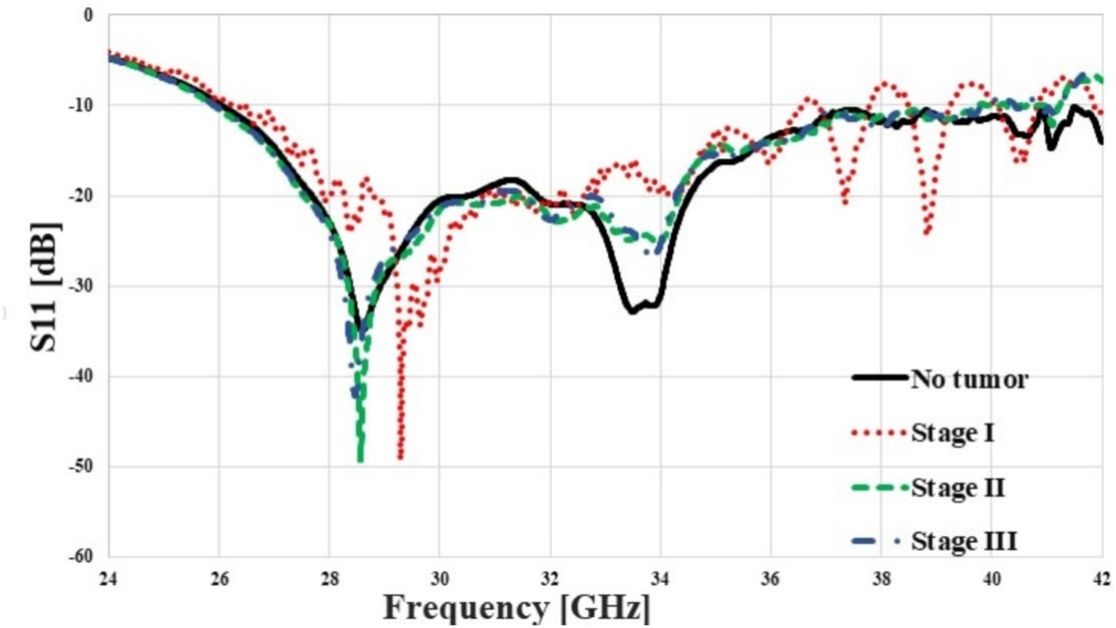
S11parameter results for tumor growing.

Because solid tumors have poor lymphatic drainage and vascular leakage, graphene-based nanomaterial’s can preferentially concentrate in tumor tissue through the increased permeability and retention (EPR) effect. Palladium can be utilized in conjunction with targeting ligands or anticancer medications for targeted therapy, and it helps make the composite more stable in the tumor microenvironment. One of the purposes of this study is to assess the impact of a polyimide-graphene composite on malignant tissues in the abdomen, including the surrounding skin, muscle layers, and subcutaneous fat. The remarkable ability of graphene-based nanomaterial’s to target and kill cancer cells through photo thermal conversion and oxidative stress generation has been shown in recent studies. By boosting the redox reaction and enhancing photo thermal stability, the addition of polyimide amplifies these benefits. Nevertheless, these nancomposites may potentially have cytotoxic or inflammatory effects on nearby healthy tissues. In order to ascertain its safety and therapeutic efficacy in the treatment of abdominal cancer, this study examines the structural and biochemical alterations in abdominal tissue exposed to polyimide-graphene, with an emphasis on oxidative stress, inflammatory biomarkers, and histopathological abnormalities^42^ .A malignant tumor inside the abdominal cavity was used to test the performance of the suggested sensor layer. Since the abdomen is a hollow area that houses a number of essential organs, including the stomach, liver, colon (large intestine), pancreas, kidneys, and the abdominal cavity itself, a realistic abdominal model was created to resemble the actual human anatomy. The tumor was tested in three different stages with varying sizes as shown in Fig. 11, to evaluate the effect of dimensional changes on the antenna performance. Table 3 lists the characteristics at of these stages. The simulated results are presented in Fig. 12. The antenna results without introducing any existing any tumor is also included in the figure for comparison.

- Stage I: The tumor was configured with size of 2.5 cm^3^ and embedded via the slice. The results showed a resonant frequency of 29.291 GHz with a reflection level of –50 dB.
- Stage II: The tumor size was increased to 9 cm^3^. The measured resonant frequency was 28.562 GHz with a reflection level of –49.105 dB.
- Stage III: A larger tumor size of 37.5 cm^3^ was evaluated, yielding a resonant frequency of 28.502 GHz and a reflection level of –42.283 dB.

**Table 3:** The three stages of abdominal cell carcinoma.

Stage	Description
Stage I	The tumor size is 2.5 cm^3^ and is confined to fat and muscle
Stage II	The tumor size is 9 cm^3^ and is confined to fat and muscle
Stage III	The tumor has spread to one or more local lymph nodes with larger size of 37.5 cm^3^, is in a vein leading from the abdomen to the heart, or has spread to the fat surrounding the abdomen or the adrenal gland

The results indicate that increasing the tumor size significantly affects the resonant frequency and reflection level, highlighting the antenna’s sensitivity to changes in the physical properties of the biological tissue. The results clearly demonstrate that increasing the tumor size significantly affects both the resonant frequency and the reflection coefficient. The results also indicate that with increasing the tumor size, a slight decrease within the frequency of resonance. This shift is attributed to the change in the overall dielectric constant of the region surrounding the antenna. As the tumor size increases, the portion with dielectric properties different from those of normal tissues becomes larger, which alters the electromagnetic field distribution and shifts the frequency toward lower values.

Additionally, it has been noted that when tumor size increases, so does the reflection coefficient (S11), showing a shift in the antenna’s impedance matching with the surrounding medium. These results show that the suggested antenna has a high sensitivity to changes in tumor size, which makes it a viable option for biological uses including growth monitoring and tumor identification and characteristics of the biological tissue. Also, the obtained results demonstrate a considerable correlation between the tumor size and the variation in the resonance frequency and reflection coefficient. Significant changes in the resonance frequency were seen as the tumor volume grew, demonstrating the impact of the malignant tissue’s shape and dielectric characteristics on the distribution of electromagnetic fields inside the abdominal cavity.

With higher reflection loss values (more negative dB), which indicate greater electromagnetic energy absorption by the tissue, the resonance frequency for lower tumor sizes stayed near that of the healthy abdomen. But as the tumor became larger, the resonance frequency gradually changed and the reflection loss decreased (smaller negative dB values). This result implies that larger tumors considerably modify the electromagnetic properties of the abdominal environment, impacting wave reflection and propagation. These results demonstrate the potential of the suggested sensing layer for medical diagnostic applications, especially in non-invasive cancer detection and monitoring systems, and validate its sensitivity in identifying alterations in tumor size within the abdominal cavity^43^ .

#### SAR assessment for biomedical applications

**Fig. 13:**
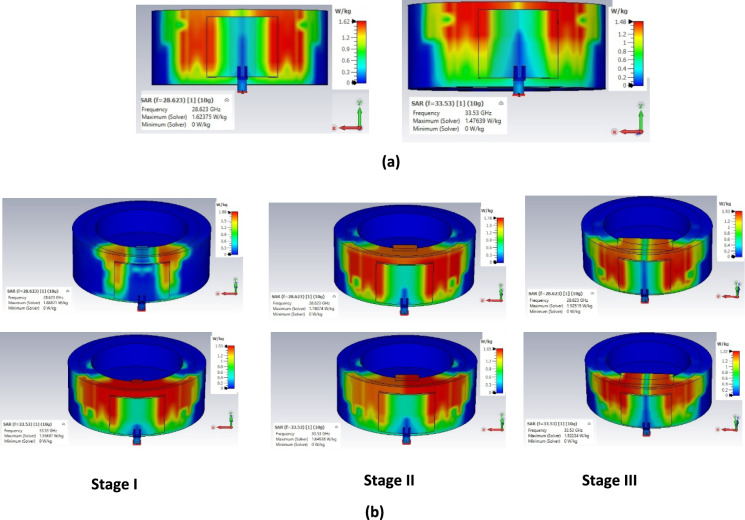
SAR distribution within the human tissue model at different operating frequencies showing increased absorption in the tumor region with all values remaining within safe limits, (**a**) with no tumor, (**b**) with tumor for the three stages.

The SAR performance of the proposed graphene–polyimide flexible antenna was evaluated to ensure compliance with international safety standards for wearable biomedical applications and the result is given in Fig. 13. Figure 13a shows the SAR distribution which is computed using a multilayer human tissue model with no tumor present at operating frequency of 28.623 GHz and 33.53 GHz. The simulation results indicate that the maximum averaged SAR over 10 g of tissue is 1.62375 W/kg and 1.476 W/kg, respectively. which is below the IEEE recommended safety limit of 2 W/kg.

Figure 13b provides the SAR distribution which computed using a multilayer human tissue model taking into account the presence of a diseased (tumor) tissue layer (three stages), to represent realistic physiological conditions, at operating frequencies of 28.623 GHz and 33.53 GHz.

The simulation results indicate that the maximum averaged SAR over 10 g of tissue reaches 1.88821 W/kg, 1.78074 W/kg and 1.52515 W/kg at frequency 28.623 GHz for stage I, II, and III respectively. While SAR reaches 1.55687 W/kg 1.64938 and 1.52234 W/kg at frequency 33.53 GHz for stage I, II, and III respectively. Despite the inclusion of the tumor tissue, all obtained values remain below the IEEE recommended safety limit of 2 W/kg, confirming also the safe electromagnetic exposure levels. The spatial SAR distribution reveals that the highest absorption occurs in the tissue region closest to the antenna surface, with a noticeable concentration in the tumor region due to its distinct dielectric properties compared to healthy tissues. This variation highlights the influence of tissue heterogeneity on SAR distribution, while the absorption gradually decreases with depth, indicating controlled electromagnetic energy penetration. These findings demonstrate that the proposed antenna maintains safe SAR levels across multiple operating frequencies even in the presence of diseased tissues, while also showing sensitivity to tissue variations. This makes the design not only suitable for on-body operation but also promising for advanced biomedical sensing and early-stage disease detection applications. Also the results demonstrate that the design is safe for on-body operation and suitable for high-frequency biomedical sensing and monitoring applications^44^.

Table 4 compares the performance of the proposed design with other recently published related works. It is found that the proposed design is very simple and provides wide bandwidth with lower SAR with higher gain.

**Table 4:** Comparison among the proposed design with other recently published related works.

Ref	Antenna Type	Active Element’s Material	Substrate’s Material	Frequency (GHz)	Gain (dB)	Return Loss (dB)	SAR (W/kg) 10 g	Application
^45^	Patch Antenna	copper	Flexible polymer	2.45	8.384	-30	1.53564	Medical
^46^	Patch Antenna	copper	Textile	2.45	4.36	-	-	Knee bone monitoring
^47^	Patch Antenna	copper	RT/Duroid	2–4	_	-31	1.646	Breast Cancer
^48^	Patch Antenna		FR4	2.4 to 2.5	1.24	− 36	-	ISM band
^49^	Rectangular, triangular, circular patches	Graphene sheet	Denim or Felt	2.443–2.464	5.89–5.98	-	0.002–0.011	Heat transfer method
^50^	Planar Inverted-F	(copper)	Textile	2.45–5.8	1.68	-19 and-26	-	Medical
^51^	Rectangular patch	Graphene Based Sheet (GBS)	Flexible	6	5.87	-	-	Molding
^52^	Multilayer patches	Graphene ink	Polyimide foam -Polyimide	3.003 5.170 6.13	2.09			Print and paste
^53^	Monopole Antenna		Flexible	5.5–7	4.6			X-band
^54^	Planar antenna	-	Textile	2.5–20	3.17	− 27		UWB Medical
Proposed Work	Flexible Antenna	Graphene	Polyimide (Kapton)	25.2–40.2	6.796	-43.2	1.62375	Wearable & Biomedical

## Conclusion

This study involved the design and implementation of a flexible graphene-based antenna that operates in the 25.2–40.2GHz frequency band. The antenna was used on biological sections of the human body, specifically the hand and the abdomen, after being independently evaluated to assess its basic electromagnetic properties. The hand model’s results demonstrated the antenna’s potent biological system interaction capabilities, which amply demonstrated the impact of electromagnetic waves on the hand tissues. Additionally, it produced good gain and reflection coefficient values, demonstrating its high efficiency in functioning in intricate biological contexts. Similar behavior in terms of contact with the abdominal biological system was observed when the antenna was applied to the human abdomen. In order to replicate actual biological settings and track tumor growth and dissemination throughout the body, a tumor was also inserted into the abdominal cavity. The simulation results showed that the antenna shows simulated sensitivity to tumor-induced dielectric changes, particularly in its advanced stages. Based on these findings, it can be said that the developed graphene-based antenna has a suggests potential that requires experimental validation applications inside the human body and has a high ability to interact with human biological systems.

## Data Availability

The datasets used and/or analyzed during the current study available from the corresponding author on reasonable request.
